# Editorial: Neurobiology of eating behavior: insights into adiposity and cardiometabolic health

**DOI:** 10.3389/fnut.2024.1448985

**Published:** 2024-07-01

**Authors:** Pey Sze Teo, Nor Azwani Mohd Shukri, Theresia Mina

**Affiliations:** ^1^Singapore Institute of Food and Biotechnology Innovation (SIFBI), Agency for Science, Technology and Research (A^*^STAR), Singapore, Singapore; ^2^Kulliyyah of Allied Health Sciences, International Islamic University Malaysia, Kuantan, Pahang, Malaysia; ^3^Nanyang Technological University Lee Kong Chian School of Medicine, Singapore, Singapore

**Keywords:** nutrition, eating behavior, epidemiology, neurobiology, cardiometabolic health, adiposity

## Introduction

Contemporary dietary trends such as intermittent fasting, ketogenic diets, or the novel it-food are popular and aplenty. Fueled by celebrity endorsements and social media, those diets are claimed to promote rapid weight loss and better health outcomes but are not necessarily backed by robust scientific evidence. The most recent headlines on the use of Ozempic by famous individuals—which directly targets appetite, a fundamental eating behavioral aspect—highlight the society's continued desire and the industry's willingness to address the market for magic weight loss solutions. Nevertheless, the long-term effect of such pharmacological agent remains unknown, and weight rebound remains an important but overlooked health issue.

The field of nutrition excels at quantifying dietary intake and evaluating the implications of these dietary choices, but there remains a significant gap in understanding the underlying factors of individual and social eating behavior. Most dietary research also hails from the Western hemisphere and may not necessarily translate well to non-Western populations due to differences in genetics, lifestyle, and cultural dietary practices. To address this, our Research Topic wanted to provide avenues for research focused on the underlying biological mechanisms of eating behavior with implications for cardiometabolic health at population levels. Here, we present a series of studies that highlight the intricate connections between eating behavior, nutrition, cognitive function, and cardiometabolic health outcomes.

## The convergence of optimal nutrition, cognitive, and cardiometabolic health

In this Research Topic, we include three population-based studies focusing on the nutritional profiles of individuals with Alzheimer's Disease (AD) (He et al.), the often-overlooked role of a micronutrient in stroke (Liu et al.), and the impact of the MIND diet (Fateh et al.), which combines elements of the Mediterranean and DASH diets on obesity and lipid profiles. The emerging evidence suggests that optimal nutrition could play a crucial role in both maintaining cognitive function, possibly delaying the onset of neurodegenerative diseases, and ameliorating lipid and adiposity risk factors for cardiovascular incidence. We chose to highlight these studies because they represent broad global populations: the NHANES study represents the western hemisphere (Liu et al.), while the AD study was conducted in China (He et al.), and the MIND diet study originates from the Kurdish populations (Fateh et al.), an underrepresented population, whose Middle Eastern diets differ from Mediterranean diets. Although future studies are required to determine the adherence and longitudinal efficacy of the MIND diet on cognitive maintenance, these studies illustrate the convergence of cognitive and cardiometabolic health and potential opportunities to concurrently address both cognitive impairment and obesity burden at population levels.

## The adaptability of eating behavior regulation

The intricacies of *in vivo* experiments could be daunting to epidemiologists, but at the heart of the two animal-based studies we included is the common goal of better understanding how eating behavior is fundamentally dysregulated under suboptimal or excess nutrition. The work by Wu et al. provides insight on how a calorie-restricting diet, which forms the core of most dietary trends, dysregulates the paraventricular hypothalamic nucleus and consequently compels our body to seek more food. Similarly, the investigation by Yan et al. implies that the constant availability of food in a modern environment might deprive us of time-restriction in feeding and reduce metabolic flexibility, leading to excess adiposity. Although animal biology may have limited translations to humans, such controlled fundamental biology experiments provide hope that the neurological regulation of eating behavior is adaptable and, if appropriately “reset” or fine-tuned, could help mitigate metabolic disorders and promote overall health. These findings support future personalized nutrition and targeted dietary interventions.

## Conclusion

The curated studies in this Research Topic provide insight into the complex interplay between eating behavior, nutrition, and health. We highlight the importance of addressing both brain and body health through nutrition, as well as the significance of understanding the underlying neurobiology of eating behaviors. As we continue to unravel the biological underpinnings of these relationships, we move closer to leveraging dietary strategies for better health outcomes and enhanced quality of life.

## Author contributions

PT: Writing – review & editing. NM: Writing – review & editing. TM: Writing – original draft, Writing – review & editing.

